# Isobenzofuranone derivative JVPH3, an inhibitor of *L*. *donovani* topoisomerase II, disrupts mitochondrial architecture in trypanosomatid parasites

**DOI:** 10.1038/s41598-018-30405-w

**Published:** 2018-08-09

**Authors:** Somenath Roy Chowdhury, Joseane Lima Prado Godinho, Jayaraman Vinayagam, Aline Araujo Zuma, Sara Teixeira De Macedo Silva, Parasuraman Jaisankar, Juliany Cola Fernandes Rodrigues, Wanderley De Souza, Hemanta K. Majumder

**Affiliations:** 1 Infectious Diseases & Immunology Division, CSIR-Indian Institute of Chemical Biology, Kolkata, 700 032 India; 2 Laboratório de Ultraestrutura Celular Hertha Meyer, Instituto de Biofísica Carlos Chagas Filho, Universidade Federal do Rio de Janeiro, Ilha do Fundão, Rio de Janeiro 21941-902 Brazil; 3 Organic & Medicinal Chemistry Division, CSIR-Indian Institute of Chemical Biology, Kolkata, 700 032 India

## Abstract

Kinetoplast DNA (kDNA) bearing unusual mitochondrion of trypanosomatid parasites offers a new paradigm in chemotherapy modality. Topoisomerase II of *Leishmania donovani* (LdTopII), a key enzyme associated with kDNA replication, is emerging as a potential drug target. However, mode of action of LdTopII targeted compounds in the parasites at sub-cellular level remains largely unknown. Previously, we reported that an isobenzofuranone derivative, namely 3,5-bis(4-chlorophenyl)-7-hydroxyisobenzofuran-1(3H)-one (JVPH3), targets LdTopII and induces apoptosis-like cell death in *L*. *donovani*. Here, we elucidate the phenotypic changes and the events occurring at sub-cellular level caused by JVPH3 in *L*. *donovani*. In addition, we have evaluated the cytotoxicity and ultrastructural alterations caused by JVPH3 in two brazilian trypanosomatid pathogens viz. *L*. *amazonensis* and *Trypanosoma cruzi*. Despite killing these parasites, JVPH3 caused significantly different phenotypes in *L*. *donovani* and *L*. *amazonensis*. More than 90% population of parasites showed altered morphology. Mitochondrion was a major target organelle subsequently causing kinetoplast network disorganization in *Leishmania*. Altered mitochondrial architecture was evident in 75–80% *Leishmania* population being investigated. Quantification of mitochondrial function using JC-1 fluorophore to measure a possible mitochondrial membrane depolarization further confirmed the mitochondrion as an essential target of the JVPH3 corroborating with the phenotype observed by electron microscopy. However, the impact of JVPH3 was lesser on *T*. *cruzi* than *Leishmania*. The molecule caused mitochondrial alteration in 40% population of the epimastigotes being investigated. To our knowledge, this is the first report to evaluate the proliferation pattern and ultrastructural alterations caused in Brazilian kinetoplastid pathogens by a synthetic LdTopII inhibitor previously established to have promising *in vivo* activity against Indian strain of *L*. *donovani*.

## Introduction

Trypanosomatid infection accounts for an alarming death tally in the continents of Asia, South America and Africa. Particularly, visceral leishmaniasis (VL), caused by *Leishmania donovani*, presents a gruesome picture in Indian subcontinent. Another clinical form, cutaneous leishmaniasis (CL), caused by *Leishmania amazonensis*, accounts for 0.7 million to 1.3 million new cases every year as per World Health Organization’s recent statistics (WHO’s fact sheet)^1^. Indian Government has taken an initiative to eliminate leishmaniasis from India by 2020. Unfortunately, to combat the disease, chemotherapy is till now the only choice^2^. To minimize host toxicity and side effects, continuous effort is being made by researchers to identify and validate novel drug targets in the parasite.

Trypanosomatid parasites possess an unusual mitochondrion which contains a structurally complex DNA known as kinetoplast DNA (kDNA). This is a planar network of maxicircles and minicircles catenated together. Absence of kDNA network in higher eukaryotes including humans has opened a new avenue in the quest of antitrypanosomatid agents which can target the kDNA. Topoisomerase II is a crucial enzyme associated with minicircle replication in the parasite^3^. Our laboratory has been engaged to explore topoisomerase II of *Leishmania donovani* (LdTopII) as a drug target for anti-leishmanial therapeutics. It is essential as well as interesting to study the effect of topoisomerase II inhibition within the parasite. Human body also has topoisomerase II. Nonetheless, if any molecule has the potential to specifically inhibit parasite topoisomerase II without hampering the host enzyme; it achieves an attribute to be a model molecule for investigation. Unfortunately, reports on parasite specific topoisomerase II inhibitors having promising *in vivo* antitrypanosomatid efficacy are substantially meagre. In an earlier study in 2014, we reported the synthesis of an isobenzofuranone derivative, namely 3,5-bis(4-chlorophenyl)-7-hydroxyisobenzofuran-1(3H)-one (JVPH3)^4^ where we established that JVPH3 is a synthetic catalytic inhibitor of LdTopII and effective to reduce the *L*. *donovani* parasite burden in an experimental model of visceral leishmaniasis (VL). However, the study was limited to a single species of *Leishmania*. A molecule qualifies to be a potential drug candidate if it can target more and more members of a pathogen family without hampering host physiology. Since trypanosomatid parasites share structural similarities in their kDNA network and have a common mechanism for kDNA replication^5^, we were interested to evaluate the effect of JVPH3 in Brazilian pathogens viz. *L*. *amazonensis* and *Trypanosoma cruzi* as well as to use the molecule JVPH3 as a model topoisomerase II inhibitor to study ultrastructural alterations caused in kinetoplastid parasites. Here we show for the first time that JVPH3 is effective to kill Brazilian strains of *Leishmania amazonensis* and *Trypanosoma cruzi*. In all *Leishmania* strains, mitochondrion was found to be substantially affected resulting in subsequent kinetoplast network disorganization. To our surprise, the phenotypic outcomes of mitochondrial targeting were significantly distinct in *L*. *donovani* and *L*. *amazonensis*. However, in *T*. *cruzi*, the outcome was somewhat different. Though the mitochondrion of *T*. *cruzi* underwent structural alteration, the kDNA topology seemed to be unaffected. Cumulatively, this report establishes the events occurring at sub-cellular level of three kinetoplastid pathogens by a parasite topoisomerase II targeted compound for the first time.

## Results

### JVPH3 changes *Leishmania donovani* cell morphology

**Figure 1:**
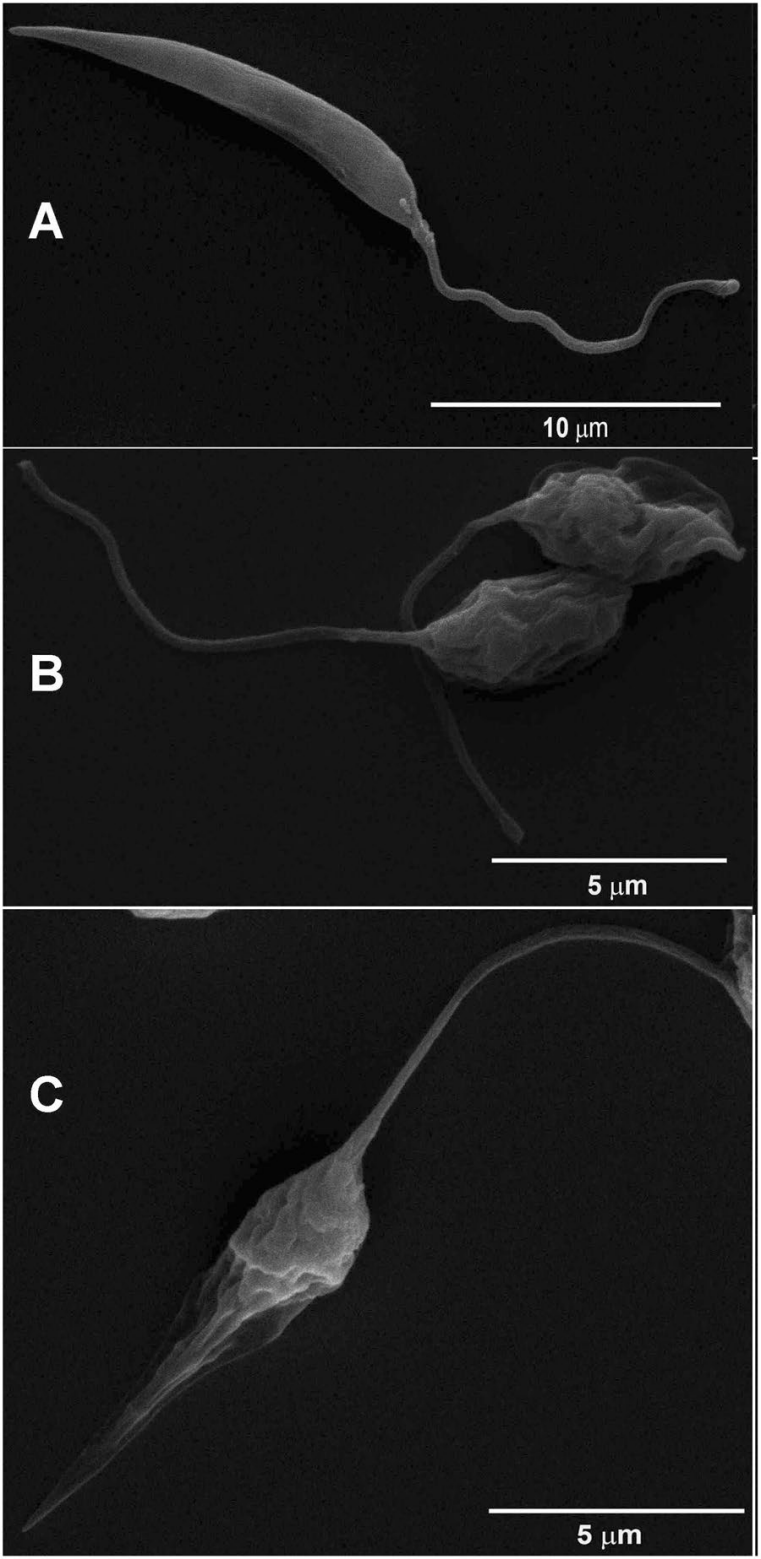
Scanning electron micrographs of *Leishmania donovani* promastigotes. (**A**) *L*. *donovani* control. (**B**) Shrunken morphology and membrane septation at 15 μM JVPH3. (**C**) Atypical phenotype at 20 μM JVPH3.

We initiated our current study by first attempting to understand the morphological changes imparted by JVPH3 in *L*. *donovani* using scanning electron microscopy (SEM). Control parasites possessed typical slender cell body with smooth cell surface and elongated flagella (Fig. 1A). Treated promastigotes exhibited shrunken morphology showing signs of multiseptation indicating a possible loss of cell volume (Fig. 1B) when treated with 15 μM of JVPH3. At 20 μM of JVPH3, the cellular materials tended to localize more towards the central portion of cell body. This was an atypical phenotype (Fig. 1C) of *Leishmania*. However, the flagellum was undisturbed (Fig. 1C). Among the population of promastigotes being observed, more than 90% showed altered morphology. Taken together, treatment with JVPH3 resulted in some unusual morphological outcomes in *L*. *donovani* never reported erstwhile.

### Mitochondria of *Leishmania donovani* is altered by JVPH3

**Figure 2:**
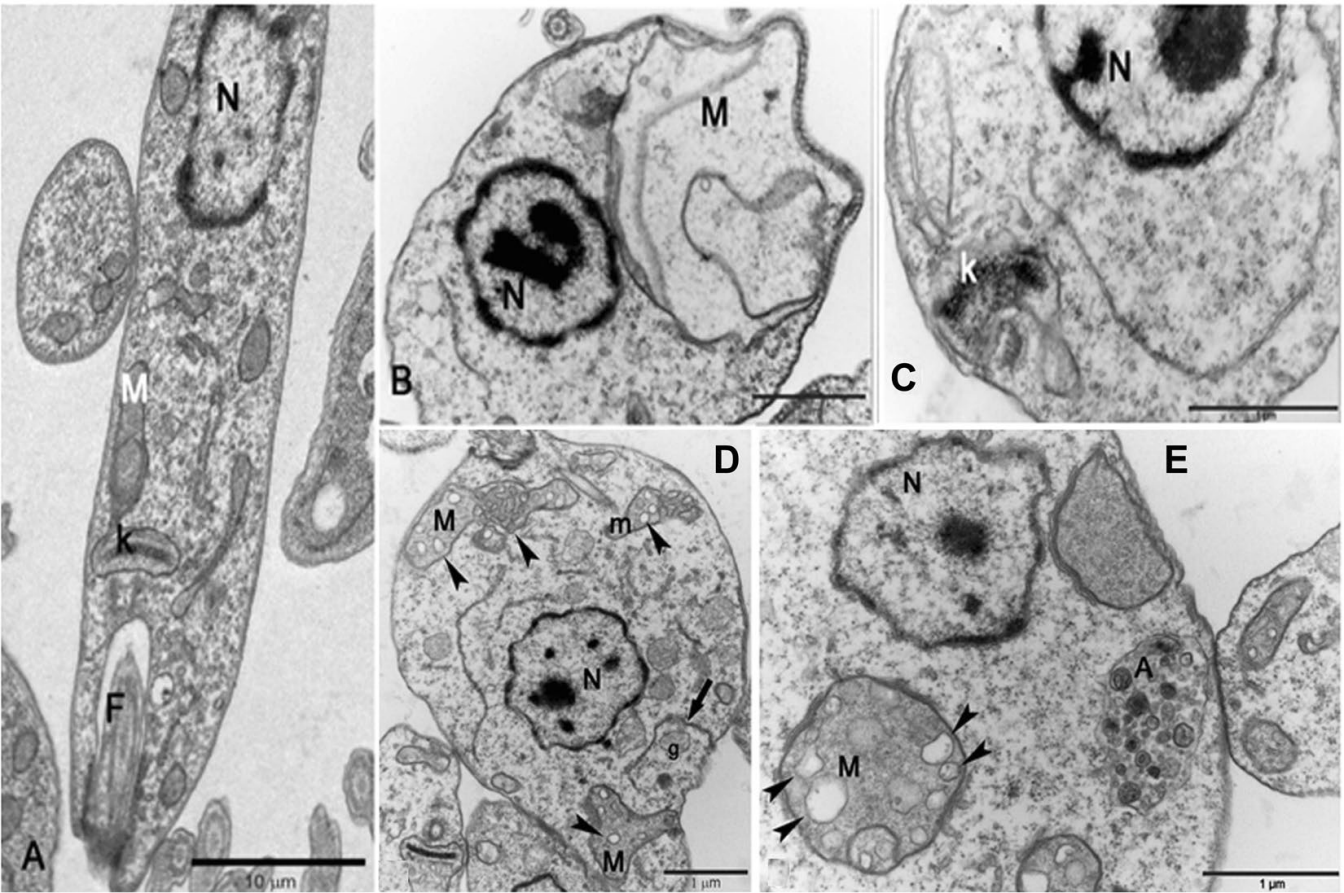
Transmission electron micrographs of *Leishmania donovani*. (**A**) General overview of control parasites presenting a ramified mitochondrion (M), kinetoplast (k), nucleus (N) and flagella (F). (**B**) Mitochondrial swelling followed by a loss of the matrix content at 15 μM JVPH3. (**C**) Disorganized kinetoplast at 15 μM JVPH3. (**D**) Intense vesiculation of the inner mitochondrial membrane (labelled as ‘M’) and phagophore formation (arrow-heads) surrounded by glycosome (g) at 20 μM JVPH3. (**E**) Presence of autophagosome containing several vesicles (labelled as ‘A’) at 20 μM JVPH3.

SEM images presented some atypical morphology in *L*. *donovani* caused by JVPH3. Though we proposed earlier an apoptosis-like death mechanism in *L*. *donovani* by JVPH3^4^, we were curious to further investigate the subcellular events occurring within the parasite. So, we performed transmission electron microscopy (TEM) to understand the alterations occurring at cell organelles. We found that mitochondria and kinetoplast structures were majorly affected in *L*. *donovani*. Being an LdTopII inhibitor, JVPH3 could be expected to impart ultrastructural alterations in mitochondria. Untreated parasites (Fig. 2A) showed normal subcellular environment. At 15 μM JVPH3, cells suffered from mitochondrial swelling followed by a loss of matrix content (Fig. 2B). Cells also showed signs of disorganized kinetoplast (Fig. 2C). These kinds of altered mitochondria including kinetoplast disorganization were evident in almost 75–80% population of the promastigotes being analyzed. At an elevated dose of JVPH3 (20 μM), signs of autophagy emerged. Some cells showed an intense vesiculation of the inner mitochondrial membrane and other phenotypes typically seen in autophagy like phagophore membrane surrounded by glycosomes and autophagosome containing vesicular structures (Fig. 2D,E). Nearly about 50% population showed these signs of autophagy.

### JVPH3 imparts cytotoxicity upon Brazilian trypanosomatids

**Figure 3:**
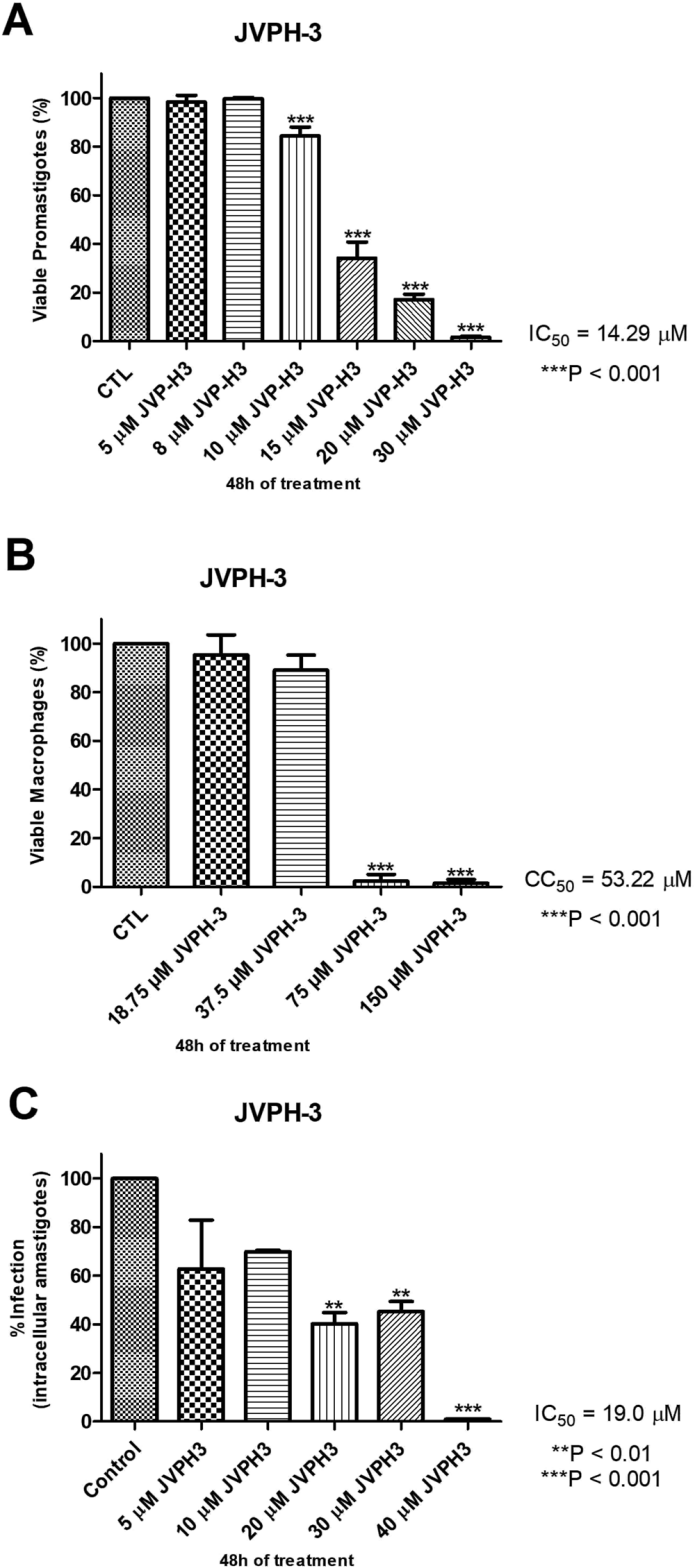
Cytotoxicity of JVPH3 in *L*. *amazonensis*. Viability of promastigotes (**A**) murine macrophages (**B**) and intracellular amastigotes (**C**) after 48 hours of treatment with JVPH3. Data of promastigote cytotoxicity assay represents the mean of at least three independent experiments; For macrophage and amastigote viabilities, number of macrophages tested are 300. P-values are based on comparison of treated groups with respect to control group (untreated promastigotes). Statistical significance of differences among the groups were assessed using the one-way analysis of variance (ANOVA) test followed by Bonferroni’s multiple comparison test in the GraphPad Prism 4 Software. Results were considered statistically significant when p < 0.05.

All the members of trypanosomatid family share significant similarity in their topoisomerase II structures. Eventually, we moved further to evaluate the effect of JVPH3 upon two other Brazilian kinetoplastid pathogens; namely, *L*. *amazonensis* and *Trypanosoma cruzi*, responsible for cutaneous leishmaniasis and Chagas disease, respectively. We found that JVPH3 killed the promastigotes of *L*. *amazonensis* in a dose-dependent manner (Fig. 3A) with an IC_50_ value of 14.29 μM at 48 hours. *Trypanosoma cruzi*, was also susceptible to JVPH3 with an IC_50_ value of 15 μM, however at 72 hours. This varying IC_50_ values accounted for differential sensitivity in two parasites. Probably due to the structural similarities of LdTopII with type II topoisomerases of these two Brazilian parasites, JVPH3 could kill those parasites by targeting respective topoisomerases. Perhaps this is the first report to ascertain the potency of an LdTopII targeted inhibitor to kill two other Brazilian kinetoplastid pathogens.

### *L*. *amazonensis* amastigote burden is reduced by JVPH3

Due to promising activity of JVPH3 against extracellular forms of Brazilian *L*. *amazonensis*, we next monitored the effect of this compound in more deadly intracellular forms. At least 300 macrophages were observed for each of the treatment. JVPH3 did not impart potential cytotoxicity upon murine macrophages (Fig. 3B). It could successfully reduce intracellular amastigote burden of *L*. *amazonensis* from murine peritoneal macrophages (Fig. 3C). The IC_50_ value was 19 μM at 48 hours.

### Ultrastructure of *L*. *amazonensis* is altered by JVPH3

**Figure 4:**
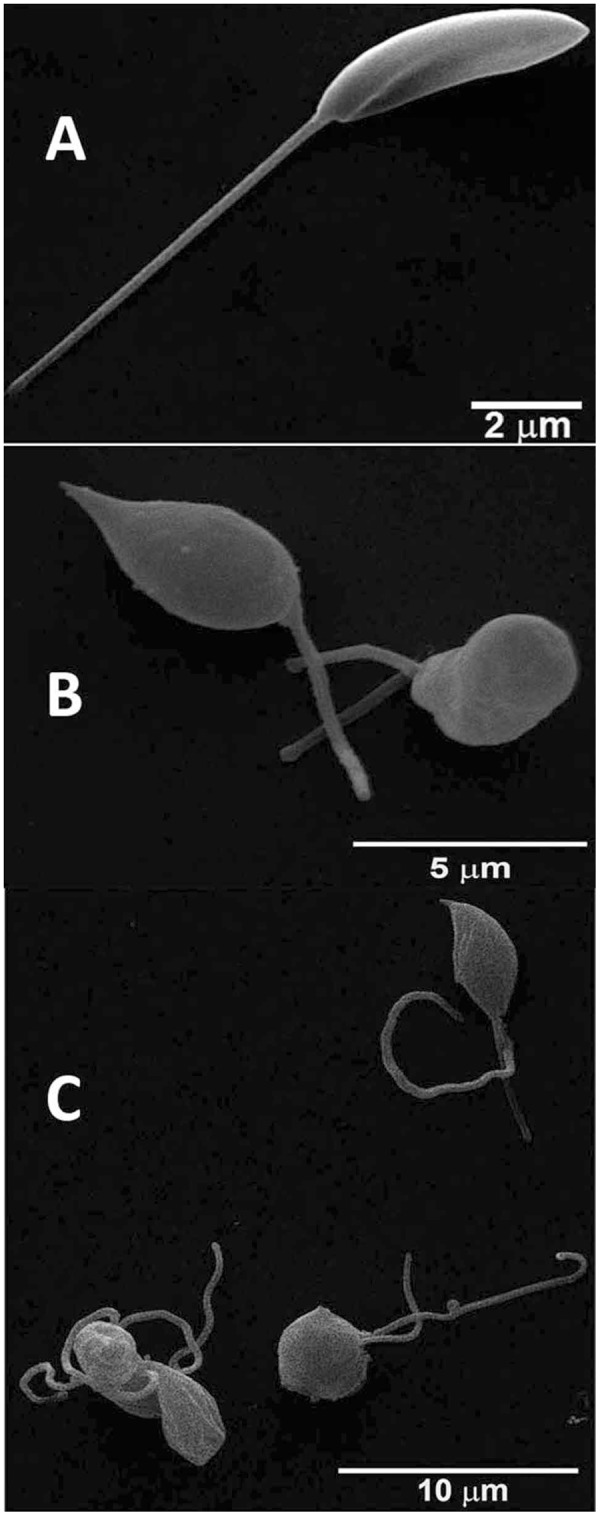
Scanning electron micrographs of *Leishmania amazonensis* promastigotes. (**A**) *L*. *amazonensis* control. (**B**) Altered morphology at 15 μM JVPH3. (**C**) Promastigotes showing more than one flagella.

**Figure 5:**
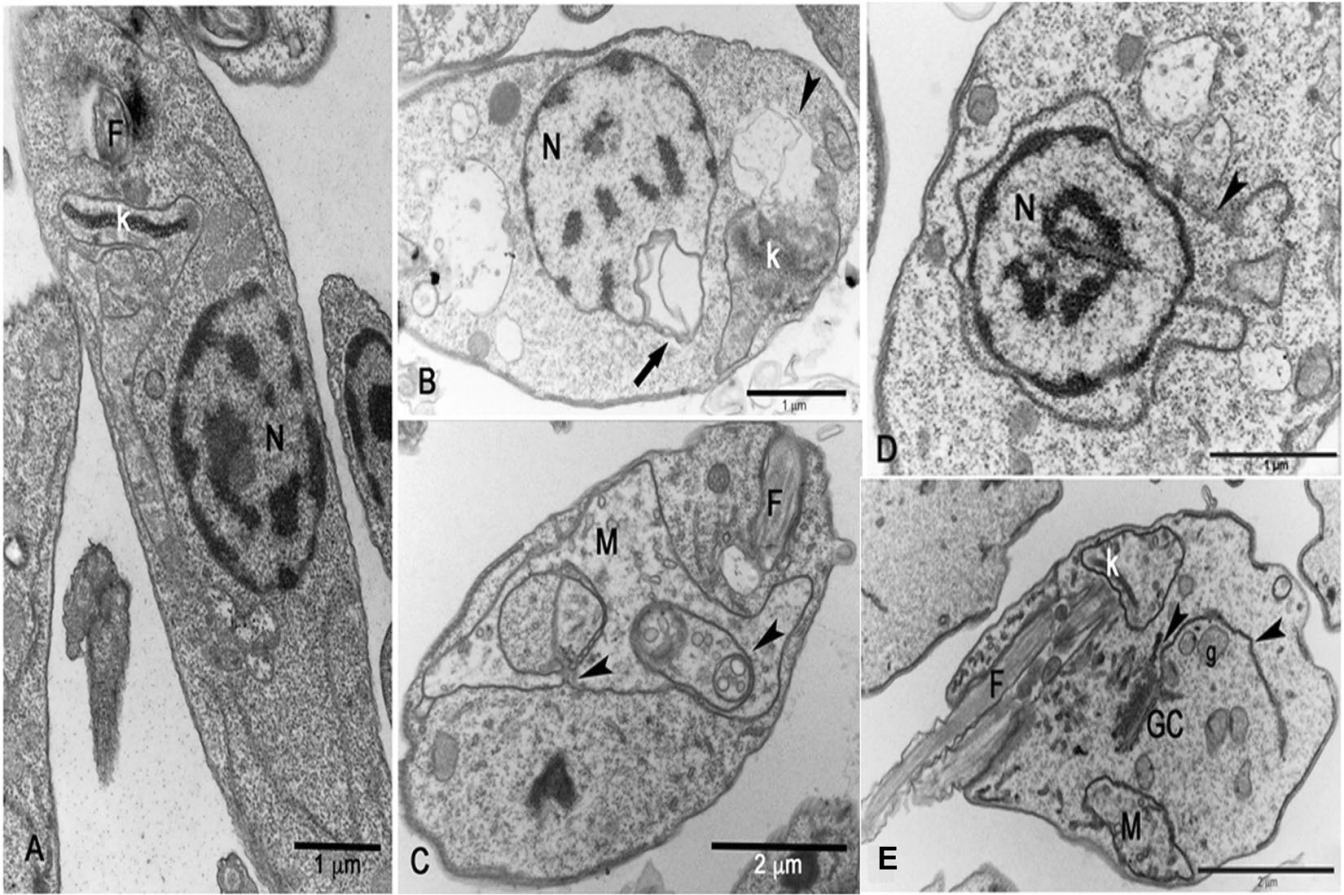
Transmission electron micrographs of *Leishmania amazonensis*. (**A**) Control (**B**) Mitochondrion of treated promastigotes (15 μM JVPH3) presented mitochondrial swelling and a complete disorganization of the mitochondrial membranes (arrowhead) resulting in the rupture of the organelle and altered nuclear membrane (arrow). (**C**) Altered mitochondrion with presence of phagophore like vesicles (arrowheads) at 20 μM JVPH3. (**D**,**E**) Abnormal distribution of endoplasmic reticulum (arrowheads) appearing in close association with organelles such as Golgi complex (GC), glycosome (g) and nucleus (N) at 20 μM JVPH3.

Since JVPH3 was cytotoxic for *L*. *amazonensis*, we hence studied the morphological changes in *L*. *amazonensis*. Surprisingly, the phenotypic outcomes were quite different. Untreated parasites showed normal morphology (Fig. 4A). At 15 μM concentration of JVPH3, cells did not show any kind of shrinkage or septation. Rather they appeared rounded and swelled mainly in the region where the nucleus was located but with homogeneous membrane surface (Fig. 4B). Many of the cells showed more than one flagellum (Fig. 4C) giving an indication of impaired cell division which might have happened due to topoisomerase II malfunctioning caused by JVPH3. Altered phenotypes were observed in more than 90% of cells being investigated. Hence, it is quite apparent that the morphological alterations were distinct in two species of *Leishmania*. However, upon evaluation with TEM, we found that mitochondria and kinetoplast structure were affected in *L*. *amazonensis* as well. Control parasites presented normal ultrastructure (Fig. 5A). JVPH3, at 15 μM concentration, caused mitochondrial swelling followed by complete disorganization of mitochondrial membrane and rupture of the organelle (Fig. 5B,C). Cells also suffered from kinetoplast disorganization. Signs with altered nuclear membrane were also identified in few parasites (Fig. 5B). Majority of the promastigotes under treatment (in the range of 80–85%) presented the ultrastructural alterations primarily in mitochondria. Similar to *L*. *donovani*, treatment with 20 μM JVPH3 resulted in abnormal distribution of endoplasmic reticulum appearing in close association with organelles like nucleus (Fig. 5D), golgi complex, and glycosome (Fig. 5E). These signs remind about autophagy process in the parasites. Ultrastructural alterations giving the indication of autophagy were evident in more than 50% population. Collectively, JVPH3 imparted different outcomes at cell surfaces of two different species of *Leishmania*. However, cellular organelles responded in similar manner.

### JVPH3 causes mitochondrial dysfunction in *Leishmania*

**Figure 6:**
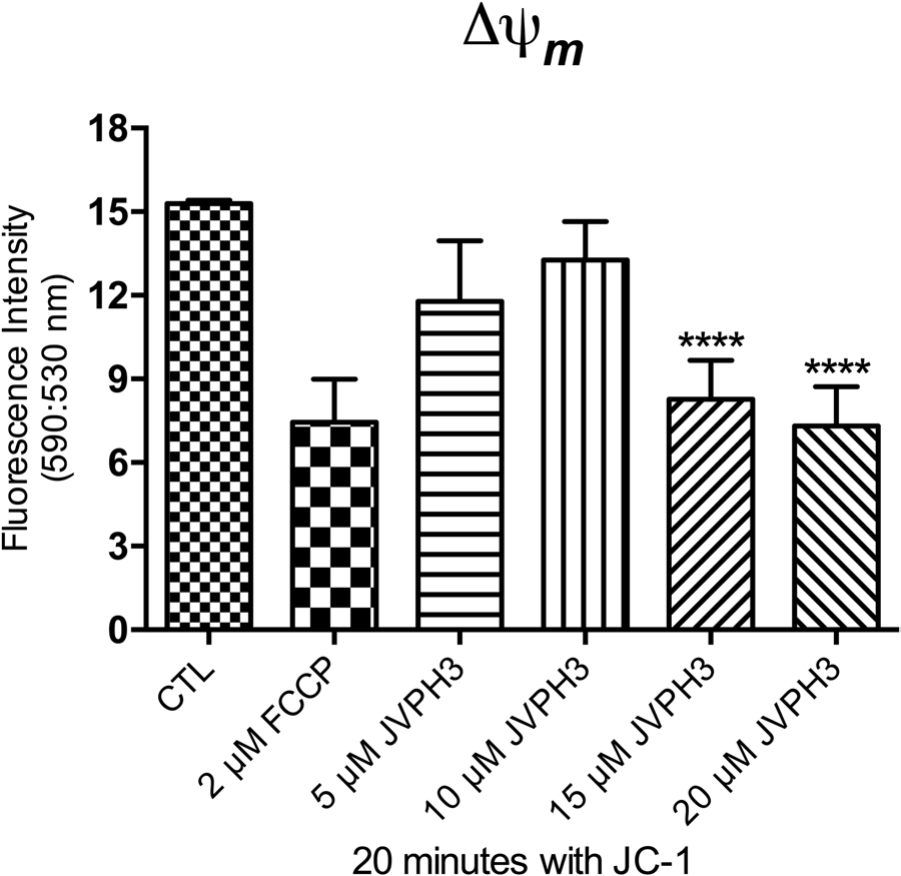
Analysis of the mitochondrial transmembrane eletric potential (ΔΨ*m*) of *Leishmania amazonensis* promastigotes after 48 h of treatment with JVPH3. ΔΨ*m* was determined by the ratio between the fluorescence intensity obtained at 590 nm (red fluorescence of energized mitochondrion) and 530 nm (green fluorescence of de-energized mitochondrion). Evaluation of ΔΨ_*m*_ after incubating the promastigotes with the respective concentrations of JVPH3. The decrease of ΔΨ_*m*_ represents the collapse in the mitochondrial transmembrane potential. 2 μM FCCP was used as a positive control to abolish the mitochondrial membrane potential. The experiments were performed in triplicate and the results are representative of them. Statistical significance of differences among the groups were assessed using the one-way analysis of variance (ANOVA) test followed by Bonferroni’s multiple comparison test in the GraphPad Prism 4 Software. Results were considered statistically significant when p < 0.05. (****P < 0.0001).

Transmission electron micrographs strongly accounted for structural alterations caused in mitochondria due to JVPH3. In order to establish the functional correlation, mitochondrial alterations were further confirmed by incubating *L*. *amazonensis* promastigotes with JC-1 fluorophore to evaluate the mitochondrial transmembrane electric potential (ΔΨ_*m*_)^6–8^. After 48 h of treatment with JVPH3, control and treated promastigotes were incubated with JC-1 for 20 min. The analysis suggested that 15 and 20 μM JVPH3 were able to decrease the ΔΨ_*m*_ (Fig. 6A). This effect was very similar with FCCP (a classical protonophore) (Fig. 6A) used as positive control. We also performed a time-dependent assay to evaluate the time dependence of mitochondrial dysfunction at different concentrations of JVPH3 (Supplementary Fig. S1).

### *Trypanosoma cruzi* presents altered mitochondria upon JVPH3 treatment

**Figure 7:**
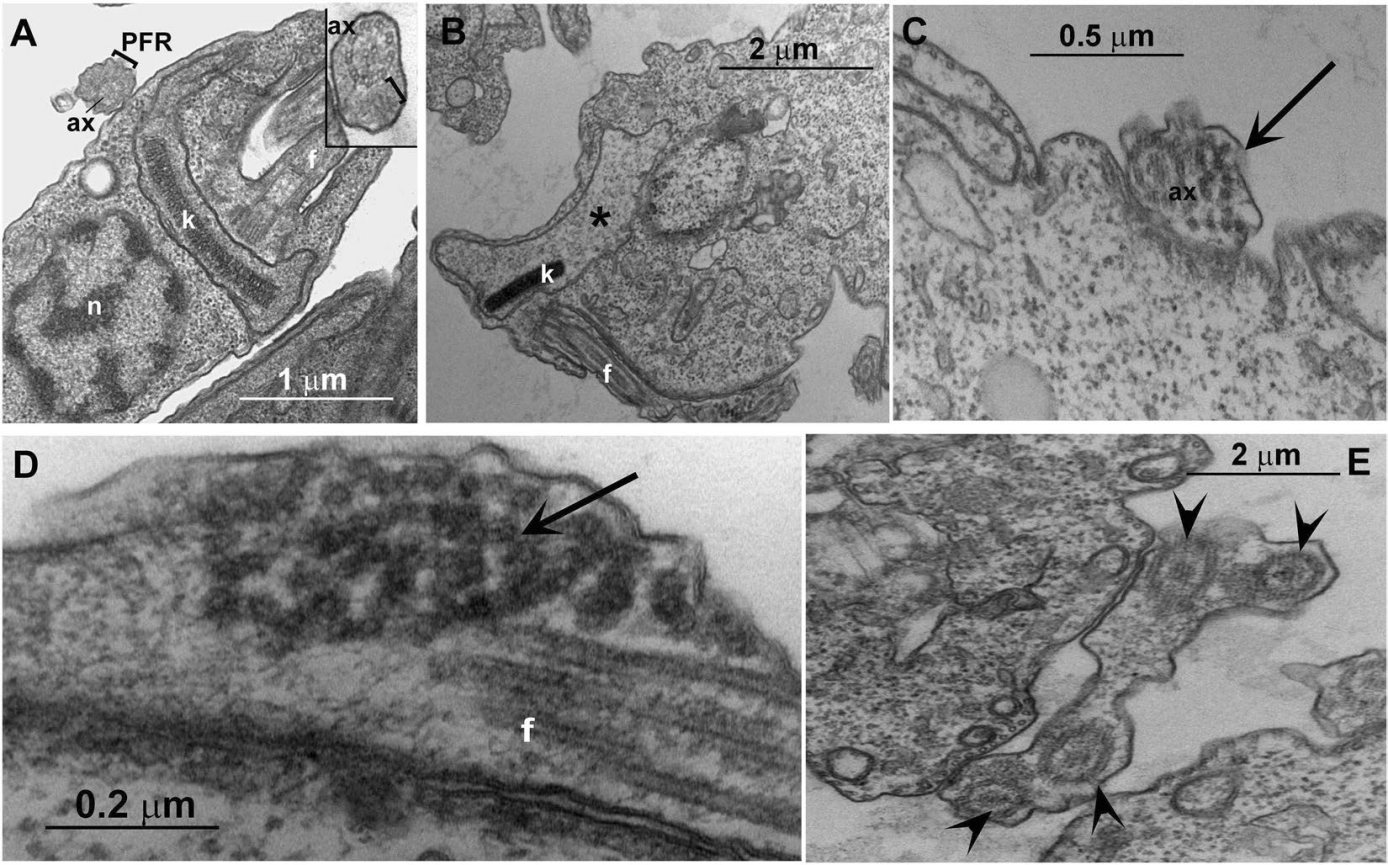
Transmission electron micrographs of *Trypanosoma cruzi*. (**A**) Non-treated epimastigote forms of *T*. *cruzi* showing the bar shape kinetoplast (k), next to the nucleus (n), and the flagellum composed by the axoneme (ax) and the paraflagellar rod (bracket). The inset highlights the axoneme and the paraflagellar rod. (**B**–**E**) Treated (20 μM) *T*. *cruzi* presented mitochondrial swelling (asterisk), a certain fragmentation of the flagellum structure (arrow) and several axonemes (arrowhead) delimited by a single membrane.

Similar to *Leishmania*, *T*. *cruzi* epimastigotes were also subjected to TEM analysis to identify the sub-cellular changes caused by JVPH3. Non-treated epimastigote forms of *T*. *cruzi* showed the bar shaped kinetoplast (k), next to the nucleus (n), and the flagellum composed of the axoneme (ax) and the paraflagellar rod (PFR) (Fig. 7A). Like *Leishmania*, *T*. *cruzi* showed mitochondrial swelling (Fig. 7B) but kDNA topology was not affected. Interestingly, flagella of treated *T*. *cruzi* underwent significant alteration. The flagella of treated protozoa presented electron dense regions which could correspond to a disorganization of the para-flagellar rod due to equivalent localization, or intra-flagellar transport (Fig. 7C,D). Moreover, we observed up to five axonemes surrounded by the same membrane (Fig. 7E), which is not typically found in *T*. *cruzi*. Among the sections being analyzed, almost 40% population presented mitochondrial swelling, 33% presented electron dense area in the flagellum and 15% presented several axonemes surrounded by the same membrane. Similar kind of ultrastructural alterations in all three trypanosomatid parasites, majorly in mitochondria, reinforced the fact that targeting topoisomerase II severely damages the mitochondrion architecture in trypanosomatid parasites. However, in case of *T*. *cruzi*, a basic difference was the unaltered kDNA topology unlike *Leishmania*. Similar to *Leishmania*, we were therefore interested to see the functional connection between ultrastructural alteration and mitochondrial dysfunctioning in *T*. *cruzi*. We hence performed mitotracker staining in *T*. *cruzi* and analyzed the treated samples both by microscopy and flow cytometry. However, there was no difference in treated parasites with respect to control cells either in fluorescence profile (Supplementary Fig. S2) or in flow cytometry based analysis (Supplementary Fig. S3). It suggested that despite causing structural disintegrity in *T*. *cruzi* mitochondria, JVPH3 did not impart significant loss of kDNA topology, dyskinetoplastidy or mitochondrial membrane depolarization in *T*. *cruzi*.

## Discussion

We previously reported that intraperitoneal administration of JVPH3 resulted in significant decrease in hepatic and splenic parasite burden in an experimental *in vivo* model of visceral leishmaniasis^4^. In order to establish a molecule as a prospective drug, it is indispensable to understand the molecular mechanism pertaining to its cytotoxic potential. In our previous study, we proposed an apoptosis-like pathway induced by JVPH3 causing topoisomerase II inhibition through biochemical studies in *L*. *donovani*. However, to our knowledge, there is no report to establish the phenotypic changes associated with LdTopII inhibition in this kinetoplastid pathogen. Even in our previous study^4^, we could not identify any remarkable change in parasite morphology by bright field microscopy. The present study establishes that how does the parasite morphology get altered when topoisomerase II is targeted. It also highlights the subcellular events associated with topoisomerase II malfunctioning. An earlier report from our laboratory established that LdTopII localizes both in nucleus and kinetoplast^9^. Since JVPH3 is a catalytic inhibitor of LdTopII, it could be expected to have effect on nucleus as well as kinetoplast architecture of *Leishmania*. Our TEM studies clearly demonstrated that JVPH3 caused severe structural disintegrity of mitochondria, and to some extent, in nucleus. However, nuclear alteration was limited to distortion in the nuclear membrane. The nuclear DNA topology was largely unaffected. Though a molecule which targets type II topoisomerase might have some impact on nuclear DNA architecture, interestingly treatment with JVPH3 did not cause remarkable alteration in the nuclear DNA architecture in any of the parasites being investigated here. Structural disorganization of kDNA could be well correlated with the problem in minicircle replication that might have caused by JVPH3. JVPH3, being an inhibitor of type II topoisomerase of *L*. *donovani*, can be expected to perturb the decatenation reaction of minicircles and maxicircles. This subsequently caused significant structural alteration in mitochondrial architecture of *Leishmania*. Our microscopic observation was further corroborated by functional studies using JC-1 fluorophore to measure a possible mitochondrial membrane depolarization. JC-1 is a lipophilic cationic fluorochrome which becomes concentrated in the mitochondrion. The dye exists as a monomer at low concentrations, where the emission is at 530 nm (green fluorescence). At higher concentrations it forms J-aggregates after accumulating in the mitochondrion and the emission is at 590 nm (red fluorescence). Thus, the fluorescence shift of JC-1 is considered as an indicator of an energized mitochondrial state giving a high precision result to measure the ΔΨ_m_ in *Leishmania*^6–8^. Collectively, the results generated from microscopy and JC-1 staining strongly account for mitochondrion to be one of the major targets of JVPH3 in the parasites.

The compound showed ultrastructural alterations in the mitochondria of *T*. *cruzi* also. But interestingly, despite causing mitochondrial swelling, the topology of kDNA was largely unaffected in *T*. *cruzi* after JVPH3 treatment. Microscopic observation using mitotracker and flow cytometry based experiments further confirmed that JVPH3 did not cause functional alteration in mitochondria or dyskinetoplastidy in *T*. *cruzi*. The mitochondrial membrane of the kinetoplast is crossed by a complex system of filaments known as tripartite attachment complex (TAC). This complex connects the kDNA to the basal body^10^. However, despite this organized and strong connection, the mitochondrial swelling does not necessarily culminate in disorganization of kDNA topology. The treatments of *T*. *cruzi* with 4-nitrobenzaldehyde thiosemicarbazone^11^, camptothecin^12^, and naphthoquinone analogues^13^ are examples of ultrastructural alterations on the mitochondrion without affecting the typical bar shape kinetoplast of *T*. *cruzi* epimastigote. Our results show that the kDNA disc was maintained even after mitochondrial swelling. This leads to the notion that perhaps a compound that targets LdTopII may or may not have the potential to target the type II topoisomerase of *T*. *cruzi* despite the structural similarities between these enzymes. Even if it targets the type II topoisomerase of *T*. *cruzi*, the phenotypic outcome might be different. Since, type II topoisomerase is indispensable for decatenating minicircles and maxicircles present in the kDNA network of *Trypanosoma* as well^3^, naturally an inhibitor which could cause type II topoisomerase malfunctioning will eventually introduce structural alteration in the mitochondria. However, this hypothesis requires further investigation because testing JVPH3 against type II topoisomerases of *L*. *amazonensis* and *T*. *cruzi* is beyond the scope of this current investigation. Considering the electron micrographs as well as functional studies, it can be ascertained that mitochondria of kinetoplastid protozoa, especially for *Leishmania* species, are one of the major targets of JVPH3. Our study also confirms that at elevated dosage of JVPH3, signs of autophagy become prominent in the parasites. Few reports suggest that *Leishmania* may undergo autophagic death as a rescue mechanism from drug induced damages^14,15^. Perhaps, when subjected to an elevated dosage of JVPH3, *L*. *donovani* turns on autophagic machineries in order to survive. TEM studies cumulatively established that when topoisomerase II of the protozoa is targeted, it potentially perturbs the mitochondrial functioning in the parasite and may eventually trigger the autophagic machineries of the cells.

Effects of many topoisomerase II targeted drugs upon trypanosomatid parasites have been evaluated. But reports on specific inhibitors of parasite topoisomerase II which do not pose adverse effect on host enzymes are significantly meagre till date. Reports suggest that prokaryotic topoisomerase II inhibitors are more effective against trypanosomatids whereas well established eukaryotic topoisomerase II targeted drug etoposide does not have significant effect upon kinetoplastid parasites^6,16,17^. We establish for the first time that JVPH3 has a distinction of being a specific inhibitor of LdTopII and effective against a wider spectrum of kinetoplastid parasites. The investigation also establishes that when topoisomerase II is targeted in the parasite, it eventually destroys the mitochondrial as well as kinetoplast architecture of the parasites. Keeping in mind the wider applicability and *in vivo* role to reduce parasite burden in mice model of VL, this molecule strongly accounts for its candidature as a potential tool against trypanosomatid infections.

## Methods

### Synthesis of 3,5-bis(4-chlorophenyl)-7-hydroxyisobenzofuran-1(3 H)-one (JVPH3)

JVPH3 was synthesized as described previously by “Mishra *et al*.”^4^. Activated I_2_ (45 mg, 20 mol%), K_2_CO_3_ (276 mg, 2 mmol/L), (E)-1,4-bis(4-chlorophenyl)but-2-ene-1,4-dione (234 mg, 1 mmol/L) or (E)-1,4-bis(4- romophenyl)but-2-ene-1,4-dione (394 mg, 1 mmol/L) and methyl acetoacetate (128 mg, 1.1 mmol/L) were added in isopropanol (10 mL) in a 50 mL flask fitted with magnetic stirrer. The reaction mixture was then stirred under reflux at 110 °C for 30 minutes. After disappearance of the starting material (monitored by thin layer chromatography) the reaction mixture was allowed to cool at room temperature. Thereafter, reduced pressure was applied in vacuum to remove the solvent. The residue was then diluted with water (10 mL) followed by extraction with CHCl_3_. The organic layer was collected, washed with brine, and then dried over anhydrous Na_2_SO_4_. Removal of solvent resulted in a solid mass which was then subjected to column chromatography over neutral alumina using petroleum ether and increasing proportion of chloroform as eluent. Petroleum ether: chloroform (70:30) eluent resulted in a solid which was recrystallized from chloroform–petroleum ether as a white solid. This solid is the compound 3,5-bis(4-chlorophenyl)-7-hydroxyisobenzofuran-1(3 H)-one (JVPH3). The purity of the compound was 98.5% as obtained by HPLC analysis (Supplementary Fig. S4)

### Parasites

Isolation and maintenance of *Leishmania amazonensis* WHOM/BR/75/Josefa strain and *Trypanosoma cruzi* Y strain were done as reported previously^18^. In brief, amastigotes of *L*. *amazonensis* was isolated from footpad nodules of infected BALB/c mice and were transformed into promastigotes in Warren’s medium containing 10% foetal bovine serum at 25 °C. For *T*. *cruzi*, epimastigote forms were grown for 24 h at 28 °C in liver infusion tryptose^18^ supplemented with 10% fetal calf serum. Promastigotes of *Leishmania donovani* WHOM/ET/1967/HU3 strain was kindly provided by the ‘*Leishmania* Collection’ of the Instituto Oswaldo Cruz and cultivated with Schneider medium containing 10% of fetal bovine serum at 25 °C.

### Electron microscopy

Promastigotes of *Leishmania* and epimastigotes of *T*. *cruzi* were fixed in 2.5% glutaraldehyde in 0.1 M cacodylate buffer (pH 7.2) and postfixed in a solution containing 1% OsO4, 1.25% potassium ferrocyanide and 0.1 M cacodylate buffer, pH 7.2. For transmission electron microscopy, cells were dehydrated in acetone and embedded in epoxy resin. Ultrathin sections were stained with uranyl acetate and lead citrate and observed under a Zeiss 900 electron microscope^16,18^. Several 2D thin sections were analyzed for each JVPH3 treatment group and untreated parasites. There were around at least 15 to 20 cells in each section. For scanning electron microscopy, promastigotes were dehydrated in ethanol, critical point-dried in CO_2_, mounted on stubs, sputtered with a thin gold layer and observed under a FEI Quanta 250 scanning electron microscope^6,18^. At least 100 cells were observed for untreated and JVPH3 treated parasites.

### Cell viability assay

Viabilities of *Leishmania* promastigotes and *Trypanosoma cruzi* epimastigotes were measured by CellTiter 96^®^ Aqueous MTS/PMS assay method (Promega) as described by “Henriques *et al*.”^19^. Promastigotes and epimastigotes, respectively, were cultured at a concentration of 1 × 10^6^ cells/ml and treated with 5, 8, 10, 15, 20 and 30 μM concentrations of JVPH3, respectively, for 48 hours. All IC_50_ values were calculated by non-linear regression using SigmaPlot Software Version 10. The reported P-values are based on comparison of treated groups with respect to control group (untreated promastigotes). Statistical significance of differences among the groups were assessed using the one-way analysis of variance (ANOVA) test followed by Bonferroni’s multiple comparison test in the GraphPad Prism 4 Software. Graphs were made by using the mean values of three independent experiments; where bars represent the standard deviation between them. Results were considered statistically significant when p < 0.05.

### *In vitro* macrophage infection

Macrophages, isolated from Balb/c mice 36–48 h post injection (intraperitoneal) with 2% (w/v) hydrolyzed starch by peritoneal lavage with ice-cold phosphate-buffered saline, were infected by *Leishmania* parasites at a ratio of 1:10 (macrophage: parasite) as reported previously^18^. When the macrophage culture reached around 50–60% infection with a number of amastigotes being 2–3 parasites per infected cell, the cells were washed with 1X PBS to remove the excess parasite in order to protect the cells from death as a consequence of parasitemia. The infected macrophages were then treated with 5, 10, 20, 30 and 40 μM concentrations of JVPH3, respectively, for 48 h. After fixing and Giemsa staining, percentages of infected cells and total number of intracellular parasites were determined by manual counting of at least 300 macrophages using light microscope. The average number of amastigotes (average of 300 macrophages counted for each experiment i.e. control and JVPH3 treatment) in control set was considered as 100% viability. Accordingly, the viabilities of amastigotes in treatment groups were calculated. Statistical significance was assessed using the one-way analysis of variance (ANOVA) test followed by Bonferroni’s multiple comparison test in the GraphPad Prism 4 Software. Bars represent the standard deviation between them. Results were considered statistically significant when p < 0.05.

### Evaluation of the mitochondrial membrane electric potential (ΔΨ) of *Leishmania* with JC-1 fluorochrome

Mitochondrial transmembrane electric potentials (ΔΨm) of *Leishmania amazonensis* control and treated promastigotes were evaluated using the JC-1 fluorochrome (Molecular Probes, United States)^6–8^. Initially, cells were washed in PBS, pH 7.2, and resuspended in a mitochondrial reaction medium containing 125 mM sucrose, 65 mM KCl, 10 mM HEPES/K+, pH 7.2, 2 mM propidium iodide (Pi), 1 mM MgCl_2_ and 500 µM EGTA. Then, 1.0 × 10^7^ parasites were incubated with 10 µg/mL JC-1 for 40 min at 25 °C, with readings made every minute using a microplate reader and spectrofluorometer SpectraMax M2/M2e (Molecular Devices). After 36 min, 2 µM FCCP was added to abolish the ΔΨm sustained at the inner mitochondrial membrane by the respiratory chain. Relative ΔΨm was obtained by calculating the ratio between the reading at 590 nm and the reading at 530 nm (590:530 ratio). Results obtained from each triplicate were analyzed and subjected to statistical analysis using Prism 4 (GraphPad software). Statistical significance was assessed using the one-way analysis of variance (ANOVA) test followed by Bonferroni’s multiple comparison test in the GraphPad Prism 4 Software. Results were considered statistically significant when p < 0.05.

### Fluorescence staining of *T*. *cruzi* mitochondria and evaluation by microscopy and flow cytometry

Non-treated and 30 µM JVPH3 treated *T*. *cruzi* epimastigotes were washed in PBS (pH 7.2) twice and incubated with 100 mM MitoTracker Red CMXRos (Thermo Fisher Scientific) for 1 hour at 28 °C. Then, parasites were washed 3 times in PBS and fixed in 2% formaldehyde in PBS for 15 minutes. For fluorescence optical analysis, the protozoa were deposited on slides coated with poly-L-lysine for 10 minutes, washed in PBS once and incubated with 1:500 4′,6-diamidino-2-phenylindole (DAPI) (Molecular Probes) for 5 minutes. After washing in PBS, the slides were mounted in ProLong Gold AntifadeMountant (Thermo Fisher Scientific) and observed using Axio Observer microscope (Zeiss). For flow cytometry analysis, after fixation parasites were resuspended in PBS and the analysis was performed using BD Accuri C6 flow cytometer considering 30000 events per sample. The data were analyzed using the BD Accuri C6 software.

### Bioethics

The experiments using BALB/c mice to isolate macrophages were approved by the Ethics Committee for Animal Experimentation of the Health Sciences Centre, Federal University of Rio de Janeiro (Protocols n. IBCCF 096/097/106), according to the Brazilian federal law (11.794/2008, Decreto no 6.899/2009). All animals received humane care in compliance with the “Principles of Laboratory Animal Care” formulated by the National Society for Medical Research and the “Guide for the Care and Use of Laboratory Animals” prepared by the National Academy of Sciences, USA.

## Electronic supplementary material

Supplementary Information
