# Supplementary material for: Isobenzofuranone derivative JVPH3, an inhibitor of L. donovani topoisomerase II, disrupts mitochondrial architecture in trypanosomatid parasites
Source: Sci Rep. 2018 Aug 9;8:11940. doi: 10.1038/s41598-018-30405-w (PMC6085290; doi:10.1038/s41598-018-30405-w)
Supplement: Supplementary file 1 — Supplementary Information [file 41598_2018_30405_MOESM1_ESM.pdf]

SUPPLEMENTARY INFORMATION

Isobenzofuranone derivative JVPH3, an inhibitor of *L. donovani* topoisomerase II, disrupts mitochondrial architecture in trypanosomatid parasites

Running title: JVPH3 disrupts mitochondrial architecture in trypanosomatids

Somenath Roy Chowdhury[1], Joseane Lima Prado Godinho[2], Jayaraman Vinayagam[3], Aline Araujo Zuma[2], Sara Teixeira De Macedo Silva[2], Parasuraman Jaisankar[3], Juliany Cola Fernandes Rodrigues[2],Wanderley De Souza[2#], Hemanta K. Majumder[1*]

[1] Infectious Diseases & Immunology Division, CSIR-Indian Institute of Chemical Biology, Kolkata 700 032, India

[2] Laboratório de Ultraestrutura Celular Hertha Meyer, Instituto de Biofísica Carlos Chagas Filho, Universidade Federal do Rio de Janeiro, Ilha do Fundão, Rio de Janeiro 21941-902, Brazil

[3] Organic & Medicinal Chemistry Division, CSIR-Indian Institute of Chemical Biology, Kolkata 700 032, India

* Corresponding author: Tel: +91 33 2499 5727; E-mail: hkmajumder@iicb.res.in

[#] Joint corresponding author: Tel: +55 21 2562 6721; E-mail: wsouza@biof.ufrj.br

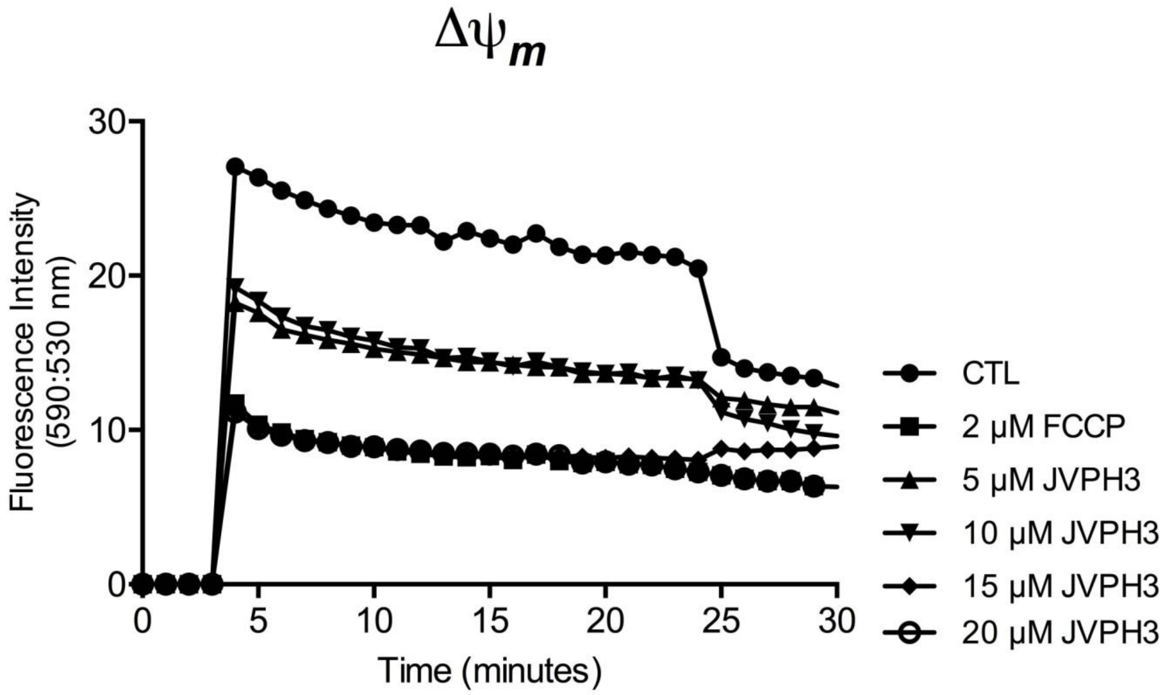

Supplementary Figure S1. Time-dependent assay indicating the significant reduction in the $\Delta\Psi_m$ at higher concentrations of JVPH3 as indicated. 2 μM FCCP was used as a positive control to abolish the mitochondrial membrane potential. ($\Delta\Psi m$ was determined by the ratio between the fluorescence intensity obtained at 590 nm (red fluorescence of energized mitochondrion) and 530 nm (green fluorescence of de-energized mitochondrion)

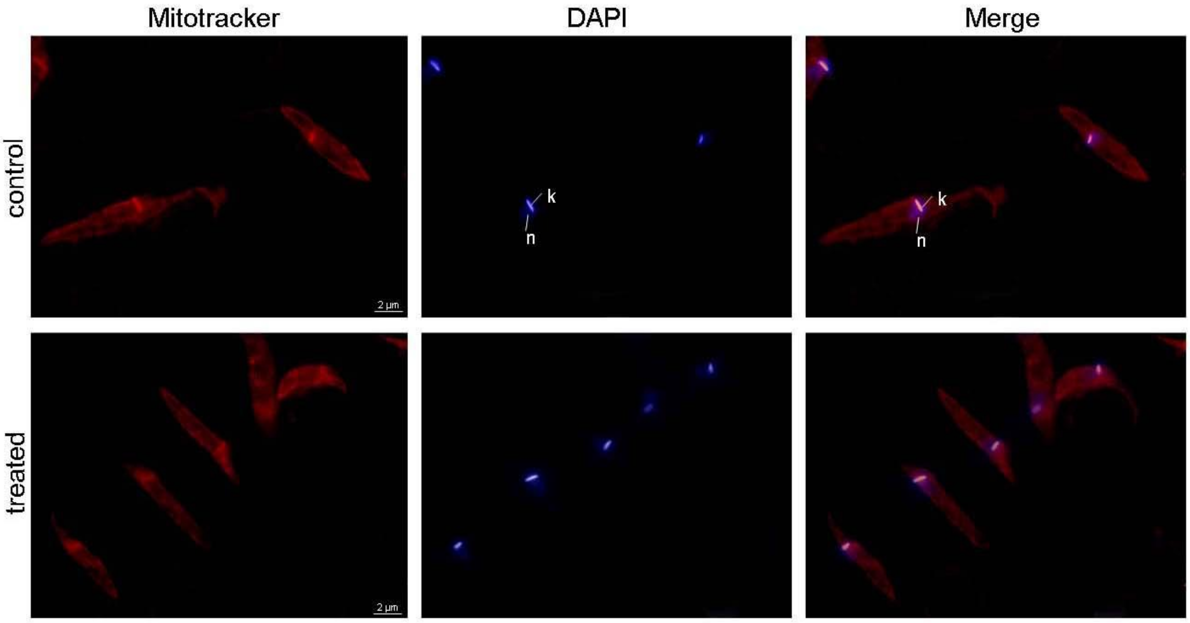

Supplementary Figure S2. Fluorescence optical microscopy of *T. cruzi* in the presence of JVPH3. Parasites were labelled with MitoTracker after 72 hours of treatment with 30 μM JVPH3. The MitoTracker labelling is distributed equivalently throughout the cell body in both treated and non-treated protozoa indicative of no mitochondrial change caused by JVPH3. Left panel –MitoTracker (Red); Middle panel – DAPI (Blue); Right panel – Merged; Upper panel – Control epimastigotes; Lower panel – Treated epimastigotes; n – nucleus; k – kinetoplast.

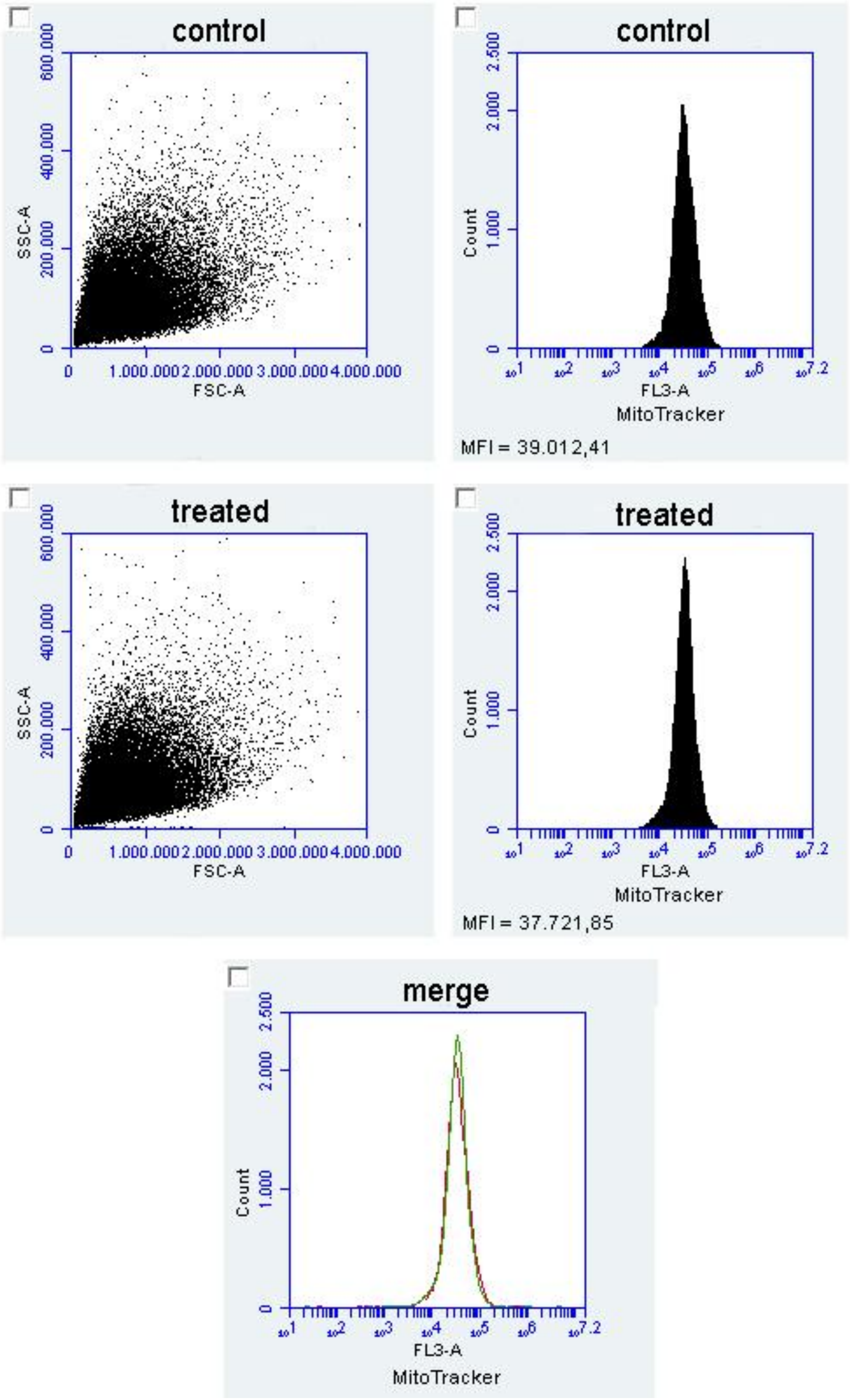

Supplementary Figure S3. Flow cytometry analysis of *T. cruzi* in the presence of JVPH3. Parasites were labelled with MitoTracker after 72 hours of treatment. The mean fluorescence intensity was similar between control and treated groups suggesting no difference in mitochondrial membrane potential. Top panel: Control cells, Middle panel: JVPH3 treated cells, Bottom panel: Merged histogram (red line represents the control group and the green one represents the treated parasites. (Experiments were done in triplicate. Data shown here is representative of one experiment)

HPLC parameters:

Column : Phenomenex Luna C18; 250mm*4.6mm*5.0µm
Mobile phase : Acetonitrile: water (70:30)
Injection volume : 20µl
Sample concentration : 1mg/ml

0

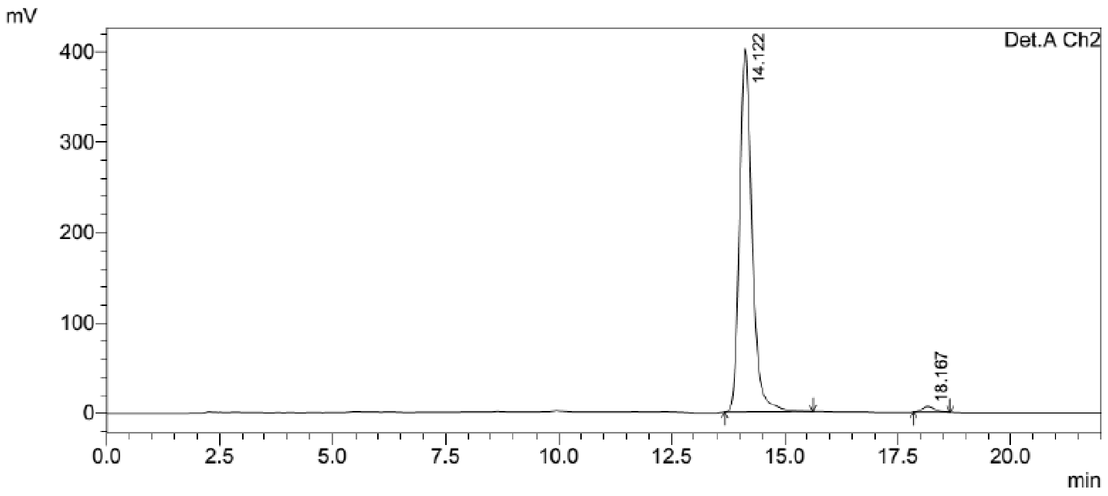

PeakTable

Detector A Ch2 254nm

Peak#	Ret. Time	Area	Height	Area %	Height %
1	14.122	7757411	402655	98.439	98.514
2	18.167	123017	6075	1.561	1.486
Total		7880428	408731	100.000	100.000

Supplementary Figure S4. HPLC analysis of the compound JVPH3.
